# Progress in targeted therapy for ankylosing spondylitis: A review

**DOI:** 10.1097/MD.0000000000040742

**Published:** 2024-11-29

**Authors:** Jiapeng Wang, Wang Lou, Yingnan Li, Yang Jiang, Xue Jiang, Lin Yang

**Affiliations:** a Department of Orthopedics, Jilin Province FAW General Hospital, Changchun City, Jilin Province, China; b Department of Anesthesiology, Jilin Province FAW General Hospital, Changchun City, Jilin Province, China; c Burn the Brotherhood of Plastic Surgery, Jilin Province FAW General Hospital, Changchun City, Jilin Province, China; d Department of Medical Laboratory, Jilin Province FAW General Hospital, Changchun City, Jilin Province, China; e Department of Rehabilitation, Jilin Province FAW General Hospital, Changchun City, Jilin Province, China.

**Keywords:** ankylosing spondylitis, biological agents, interleukin-23, interleukin-17, targeted therapy, tumor necrosis factor

## Abstract

Ankylosing spondylitis (AS) is a chronic inflammatory disease characterized by axial osteoarticular inflammation and tendon enthesitis with unclear pathogenesis. Nonsteroidal anti-inflammatory drugs and antirheumatic drugs used in the traditional treatment of AS have some problems such as drug intolerance and inadequate treatment response. Since the introduction of biological agents in the treatment of AS, they have completely changed the treatment concept of AS, and because of their safety and good tolerance, they have become the main choice for clinical AS patients. This article systematically summarizes the current status of targeted therapy for AS worldwide, analyzes the advantages and disadvantages of different types of biological agents in the treatment of AS, and provides an objective evaluation of clinical targeted therapy for AS, in order to provide a new perspective for clinical standardized treatment.

## 1. Introduction

Ankylosing spondylitis (AS) is a chronic inflammatory rheumatic disease with a genetic predisposition, which is characterized by inflammatory pain in the axial and sacroiliac joints, structural damage of bone and joint, and pathological new bone formation.^[1]^ The patients are mainly young adults aged 20 to 30 years old, and the patients are prone to spinal joint fusion when the disease progresses to the end. At the same time, more than half of AS patients have peripheral joint involvement, accompanied by extra-articular diseases, such as acute uveitis and osteoporosis.^[2,3]^ The pathogenesis of AS is still unclear. At present, nonsteroidal anti-inflammatory drugs (NSAID), corticosteroids and biological agents are used in the clinical treatment of AS. NSAID, AS the first-line drug for AS, can relieve the pain of AS patients, but the effect of improving disease activity and delaying disease progression is not ideal. About 1 third of patients will suffer from drug intolerance, and some patients will suffer from aggravation of symptoms, infection, hematological abnormalities, gastrointestinal reactions and severe liver and kidney injury.^[4,5]^

In recent years, since the introduction of biological agents into the treatment of AS in the long treatment cycle, it has completely changed the treatment concept in the field of rheumatology and has become a common clinical drug for AS patients, which is also a milestone progress in the treatment of AS. However, we still need to further explore how to rationally select drugs for AS patients in clinical practice, to clarify the advantages and disadvantages of different types of biological agents in the treatment of AS, and to develop safe and reliable individualized drug administration regimens for AS patients. At present, there is a lack of systematic summary of different types of biological agents for the treatment of AS. In this paper, Tumor Necrosis Factor-α (TNF-α) inhibitors, interleukin-17 (IL-17) antagonists and IL-12/23 antagonists used in the targeted therapy of AS have shown certain advantages in the treatment of AS (Table 1). Clarifying the issues that need to be considered in the treatment of different types of drugs provides a reasonable reference for the targeted therapy and drug selection of AS.

**Table 1:** Summary of the different types of targeted therapy drugs for ankylosing spondylitis.

Type	Drugs	Characteristics of drug action	References
Antirheumatic drugs targeting TNF-α	Etanercept	Restructuring, dimers fusion protein, soluble TNF alpha receptors, the combination of soluble and cell and inactivated TNF and lymphocyte toxins alpha, block TNF alpha with the combination of the cell surface of TNF alpha receptors.	^[11]^
	Infliximab (IFX)	A human-mouse chimeric monoclonal antibody against TNF-α synthesized in vitro can bind to TNF-α and block its biological activity.	^[16]^
	Adalimumab (ADA)	Fully human, recombinant monoclonal antibody against TNF-αIgG1.	^[22]^
	Golimumab (GDM)	The humanized monoclonal antibody anti TNF alpha drugs, targeted and soluble active forms of TNF alpha and across membrane, prevent its and TNF receptor, thus inhibiting TNF biological activity.	^[30]^
	Certolizumab pegol (CZP)	Monoclonal antibody to TNF-α humanized Fab fragments with polyethylene glycol.	^[33]^
Antirheumatic drugs targeting IL-17	Secukinumab	Fully human monoclonal antibody against IL-17A.	^[42]^
	Ixekizumab (IXE)	A high-affinity monoclonal antibody targeting IL-17A.	^[45]^
	Bimekizumab	A novel humanized IgG1 monoclonal antibody can selectively bind to specific sites on IL-17A and IL-17F, and prevent IL-17A and IL-17F from binding to their receptors.	^[48]^
	Brodalumab	Anthropogenic IgG2 monoclonal antibodies, can be combined with a selective IL-17 receptors, inhibit its and cytokine IL-17 A, IL-17 F, IL-17 c, IL-17 A/F heterodimer interaction and IL-25.	^[51]^
	Netakimab (NTK)	A human monoclonal antibody targeting IL-17A.	^[53]^
Antirheumatic drugs targeting IL-12/23	Ustekinumab (UST)	Fully human IgG1 monoclonal antibody targeting the p40 subunit of IL-12/23 interaction.	^[58]^
	Risankizumab	Humanized monoclonal antibody targeting the p19 subunit in IL-23.	^[60]^
Antirheumatic drugs targeting IL-1	Anakinra	Sufficient non-glycosylated IL-1R antagonist can block the binding of IL-1α and IL-1β to the receptor.	^[65]^
Antirheumatic drugs targeting IL-6	Tocilizumab (TXZ)	Synthetic monoclonal antibody against the IL-6 receptor.	^[68]^
Antirheumatic drugs targeting leukocyte differentiation antigens	Rituximab (RTX)	Direct effects on B cell surface molecules of CD20 monoclonal antibodies.	^[72]^

## 2. Antirheumatic drugs targeting TNF-α

Chronic inflammation and heterotopic ossification are the main features of AS that distinguish it from other rheumatic diseases. Mesenchymal stem cells (MSC) are the main source of osteoblasts and the bridge between bone metabolism and immune homeostasis.^[6]^ TNF has activated lymphocytes and osteoclasts, and promote the role of angiogenesis, the AS-MSC induction of TNF alpha mediated local inflammation, osteogenesis differentiation to enhance and lead to pathological osteogenesis.^[7]^ TNF inhibitor (TNFi) can relieve joint inflammation, pain and damage in AS patients by blocking key proteins in the inflammatory chain reaction.^[8]^ At the same time, TNFi has a rapid clinical effect and can delay bone destruction in AS patients, thereby delaying the imaging progress of AS patients. It has been widely used in clinical practice and has shown good therapeutic effects.^[9]^ Adverse reactions of TNFi include allergic reactions, infection (such as tuberculosis and hepatitis B activation), and the risk of inducing autoimmune diseases and tumors. It is necessary to carefully evaluate the patient’s condition before medication and closely monitor the patient’s changes during medication to ensure the safety of medication.^[10]^

### 2.1. Etanercept

Etanercept is a recombinant, dimeric, soluble TNF-α receptor fusion protein that is linked from the extracellular portion of the TNF-α receptor of human P75 to the Fc portion of human type 1 immunoglobulin (IgG1). By binding and inactivating soluble and cell-bound TNF and lymphotoxin α, the binding of TNF-α to TNF-α receptor on cell surface is blocked, and the abnormal immune response and inflammatory process mediated by TNF-α receptor are inhibited.^[11]^ Etanercept was approved by the FDA and EMA in 1998 and 2000, respectively, for the treatment of AS, psoriatic arthritis, rheumatoid arthritis and juvenile idiopathic arthritis. Etanercept entered Japan in 2005 and China in 2010 under the trade name Enbrel. Compared with traditional drugs, etanercept has rapid onset, obvious effect, mild adverse reactions and good tolerance.

Jennifer^[12]^ included 40 AS patients in the study and randomly divided them into the placebo group and the etanercept treatment group. During the treatment period, after 4 months of continuous treatment, 80% of the patients in the etanercept group had treatment response, while only 30% of the patients in the placebo group had treatment response (*P* = .004). Etanercept was well tolerated, and there was no significant difference in the incidence of adverse events between the 2 groups.

About TNFi structural lesions in patients with the AS information is limited, the influence of Maksymowych^[13]^ to compare in clinical trials in accordance with the heap treated patients with recent onset AS the sacroiliac joint of nuclear magnetic resonance imaging (MRI) 2 years structural changes and pathological changes. To evaluate the relationship between the disease activity score (ASDAS) of AS and the changes of MRI structural parameters. Study included 163 patients from EMBARK test (etanercept) and 76 patients from DESIR test (placebo). Compared with the control group, the proportion of patients with reduced sacroiliac joint erosion and increased backfilling under MRI in the etanercept group was significantly higher. ASDAS continued after the use of etanercept was 32.7% (34/104) of patients in remission and didn’t get ASDAS compared to continue to ease the patients (5/104), erosion decrease and the proportion of backfilling increase significantly higher (*P* < .001), but it continues to ease and the connection between the MRI lesions structural changes is unclear. Anti TNF-α drugs etanercept therapy of patients in the first 6 months of treatment significantly increased the risk of serious infection, the most common adverse events are nasopharyngitis (15%) and upper respiratory tract infection (6%), the AS patients received etanercept treatment should be done before the tuberculosis screening, infection, malignancy, and demyelinating disease prevention measures.^[14]^

Benepali (SB4), a biosimilar of etanercept, has been approved by the European Union for all adult indications of etanercept, including rheumatoid arthritis, axial spondyloarthritis, psoriatic arthritis, etc. SB4 is well tolerated and has similar safety profile to etanercept, and its pharmacokinetic properties and long-term efficacy in healthy volunteers are equivalent to etanercept. SB4 has become an equivalent treatment alternative for adult patients with autoimmune inflammatory diseases requiring etanercept treatment.^[15]^

### 2.2. Infliximab

Infliximab (IFX) is the in vitro synthesis of TNF-α human-mouse chimeric monoclonal antibodies, can be combined with TNF-α and blocking its biological activity.^[16]^ IFX respectively in 1998 grade 1999 approved by the FDA and the EMA, used in the treatment of the AS, rheumatoid arthritis, psoriasis, and crohn’s disease and the treatment of the disease such as ulcerative colitis. In 2007, IFX entered the Chinese market under the trade name of Remicade, which was also the earliest biological agent used in the clinical treatment of AS. In a study evaluating the efficacy of IFX in patients with AS, 201 patients received 5 mg/kg IFX and 78 patients received placebo. After 24 weeks, 61.2% of AS patients in the IFX group achieved symptom relief, compared with 19.2% of those in the placebo group (*P* < .001). In the IFX group, clinical benefits were observed in the second week and maintained during the 24-week study.^[17]^

In a 3-year extension study of IFX, it was shown that the improvement of symptoms in AS patients in the first and second years persisted in the third year, and during the study period, there were no related side effects in AS patients, and no drug withdrawal events occurred due to drug-related adverse events.^[18]^ An 8-year long-term study by Baraliakos^[19]^ pointed out that the early clinical response to IFX treatment could predict the long-term outcome of AS patients. Nearly half of the AS patients in the study completed the 8-year long-term follow-up, and 90% of them were in partial remission and had significantly reduced disease activity.

IFX can not only improve the AS patients with clinical symptoms, but also can prevent joint destruction, when disease activity decrease, but stopping IFX, but in the use process appears to be vigilant IFX adverse reactions such AS the most common infections and transfusion reaction.^[20,21]^

### 2.3. Adalimumab

Adalimumab (ADA), a fully human, recombinant monoclonal antibody acting on TNF-αIgG1, was approved by FDA and EMA in 2002 and 2003, respectively, for the treatment of AS, rheumatoid arthritis, psoriatic arthritis and ulcerative colitis. ADA in 2010 to enter the Chinese market, commodity name Humira, its safety and effectiveness has been confirmed.^[22]^

A 24-week, multicenter, randomized, double-blind, placebo-controlled study^[23]^ found that at week 12, 58.2% of AS patients who received subcutaneous injections of ADA40 mg every other week had a clinical remission rate, compared with 20.6% of those who received placebo (*P* < .001). ADA treatment achieved partial remission, but the incidence of adverse events was 75% in ADA treatment group and 59.8% in placebo treatment group (*P* < .05). At the same time, studies have found that ADA treatment can improve the AS patients with intestinal flora, Comamonas in abundance, this also means that gut microbes or license AS a potential biomarker assessment AS treatment.^[24]^

To determine the safety and efficacy of alternating ADA with etanercept in patients with AS, in an open-label, randomized, controlled, crossover clinical trial in patients with active AS, patients were randomly assigned to 2 sequential groups: etanercept group 1 (treatment group) or ADA group 1 (control group) for 8 weeks, followed by an additional 8 weeks of switching. Two groups of patients in 8 weeks assessment in ankylosing spondylitis (ASAS) scale improvement (ASAS20) response rate was 33% and 20% and 44% (*P* = 1.000); The ASAS40 response rates were 22% and 22%, respectively (*P* = 1.000), which further proved the equivalence of these 2 drugs in the treatment of AS, and these 2 groups were well tolerated, and no serious adverse events occurred.^[25]^ At 2-year follow-up, it was found that AS patients treated with etanercept did not differ significantly from those treated with ADA in terms of disease activity, and AS patients treated with etanercept had a higher survival rate compared with AS patients treated with ADA.^[26]^ Studies on the long-term safety of ADA found that serious infection events were the most commonly reported serious adverse reactions, but their incidence in AS patients was only 2%.^[27]^

GP2017^[28]^ and FKB327^[29]^ are biosimilar drugs of ADA, which have been approved by the European Union for all indications of ADA. They have similar physical and chemical properties and functional characteristics with ADA. BAT1406 as biological analogs of ADA, in 2019 China listed, commodity called music stand, this is the first listed ADA analogues in China.

### 2.4. Godamumab

Golimumab (GDM) is a fully human anti TNF-α monoclonal antibody drug that targets to neutralize soluble and transmembrane active forms of TNF-α, preventing it from binding to TNF receptors, thereby inhibiting the biological activity of TNF. Compared with other anti TNF-α drugs, GDM requires less frequency of administration, especially for AS patients who have received other TNF-α treatment and drug-resistant AS patients.^[30]^

The degree of humanization of GDM is up to 99.1%, and in the standard treatment of AS, only subcutaneous injection is needed every 4 weeks. Studies have found that GDM is more effective for patients with nonimpact spondyloarthritis who have high C-reactive protein (CRP) levels and/or positive MRI results.^[31]^ In a 16-week, randomized, double-blind, placebo-controlled study,^[32]^ 197 patients were randomized to receive either GDM (n = 97) or placebo (n = 100). ASAS20 remission at week 16 was compared between the GDM and placebo groups. The remission rate of ASAS40 in GDM group was 56.7%, which was significantly higher than 23.0% in placebo group (*P* < .0001).

### 2.5. Cetuzumab

Certolizumab pegol (CZP) is a polyethylene glycol humanized fragment crystallizable (Fab) fragment of TNF-α monoclonal antibody,^[33]^ which was approved by FDA and EMA in 2008 and 2009 respectively. Used in the AS, crohn’s disease,^[34]^ gestational diseases such AS psoriasis^[35]^ treatment, approved in 2019 to enter the Chinese market, commodity called Cimzia.

In a phase 3 study of CZP,^[36]^ patients were randomly assigned in a 1:1:1 ratio to receive 200 mg of CZP every 2 weeks, 400 mg of CZP every 4 weeks, or placebo. At week 12, the ASAS20 remission rate was observed. The ASAS20 remission rate was 57.7% in CZP200 mg every 2 weeks group, 63.6% in CZP400 mg every 4 weeks group, and 38.3% in placebo group (*P* < .001). In a 96-week phase III randomized trial,^[37]^ clinical improvement at week 24 was sustained to week 96 with both CZP dosing regimens, and patients in both groups had a favorable safety profile. A study of 11,317 patients who underwent CZP summary analysis of clinical trial data found that infection, malignancy, autoimmune/hypersensitivity events is still CZP therapy in patients with the most common serious adverse events.^[38]^

## 3. Antirheumatic drugs targeting IL-17

IL-17 mainly secreted by activated Th17 lymphocytes, Th17/ IL-17 play an important role in the AS inflammation of the axis, IL-17 can be direct or indirect inhibition of chondrocytes and osteoblasts in the matrix, through the induction of the matrix metal protein and the production of nitric oxide synthase, which involved in cartilage destruction process. IL-17 can interact with epithelial cells, endothelial cells, fibroblasts, monocytes and dendritic cells to promote the release of inflammatory factors such as IL-1, TNF and IL-6, and produce synergistic effects to amplify inflammation.^[39]^ AS a chronic and progressive inflammatory disease, the serum level of IL-17 in patients with AS is higher than normal,^[40,41]^ which leads to the abnormality of IL-1, IL-6, IL-8, TNF and other inflammatory factors, making the inflammation of AS recurrent, aggravating the inflammatory bone destruction, speeding up the process of bone destruction and new bone formation, and promoting the progress of AS.

### 3.1. Secukinumab

Secukinumab, a fully human monoclonal antibody directed against IL-17A, is the most characterized member of the IL-17 ligand family. AS a first-class IL-17 inhibitor, Secukinumab was approved in the United States and Europe in 2016 for the treatment of diseases such as moderate to severe psoriasis, active AS, active psoriasis-arthritis, and axial spondyloarthritis.^[42]^

In a phase III randomized trial of Secukinumab in patients with active AS,^[43]^ 371 patients were randomly assigned in a 1:1:1 ratio to different treatment groups. In group I, patients received 10 mg/kg IV at baseline and weeks 2 and 4, followed by 150 mg subcutaneously every 4 weeks; Patients in group II received intravenous injection of 10 mg/kg at baseline, weeks 2 and 4, and then subcutaneous injection of 150 mg once every 4 weeks. Group III was given placebo. At week 16, there was statistically significant difference in BASDAI improvement between groups I and II compared with placebo (−2.3 vs −0.6 for both regimens; *P* < .0001). Studies have found that the safety of Secukinumab is good, and latent pooled infection and combined reactivation are not common, which is suitable for the use of Secukinumab in chronic systemic inflammatory diseases. Secukinumab is usually well tolerated during 5 years of treatment, the most commonly reported adverse events is nasopharyngitis, Secukinumab is treating unused TNF inhibitors in patients with active AS an effective therapy, inadequate response to TNF inhibitors or intolerance patients provides a useful therapeutic option.^[44]^

### 3.2. Ixekizumab

Ixekizumab (IXE), a high-affinity monoclonal antibody targeting IL-17A, was approved by the FDA and EMA in 2016 for the treatment of ankylosing spondylitis and plaque psoriasis.^[45]^

In a phase 3 randomized, double-blind, controlled trial between June 20, 2016 and August 22, 2017, 341 patients were randomly assigned to placebo (n = 87), ADA (n = 90), IXEQ2 (n = 83), or IXEQ4 (n = 81). At week 16, 18% of placebo patients achieved ASAS40 and 52% of IXEQ2 patients achieved ASAS40 (*P* < .0001).^[46]^ The efficacy and safety of IXE in the treatment of AS patients have been confirmed, especially for patients who are intolerant to TNFi, the use of AS treatment can achieve breakthrough progress, but IXE is not suitable for AS patients with inflammatory bowel disease and uveitis, and patients receiving long-term IXE treatment should pay attention to prevent Candida infection.^[47]^

### 3.3. Bimekizumab

Bimekizumab is a new humanized immunoglobulin G1 monoclonal antibody, which can selectively bind to specific sites on IL-17A and IL-17F, prevent IL-17A and IL-17F from binding to the receptor, and thus inhibit the activation of a series of inflammatory responses in the IL-17 pathway.^[48]^ In 2 parallel phase 3 randomized controlled trials,^[49]^ 47.7% of AS patients treated with 160 mg bimekizumab every 4 weeks achieved ASAS40 at week 16, while only 21.4% of AS patients treated with placebo achieved ASAS40 (*P* < .001).^[40]^ Compared with placebo, the most common adverse reactions in the bimekizumab group included nasopharyngitis, upper respiratory tract infection, pharyngitis, diarrhea, headache, and oral Candida infection.^[50]^

### 3.4. Brodalumab

Brodalumab is A human immunoglobulin 2 (IgG2) monoclonal antibody that selectively binds to human interleukin-17 (IL-17) receptor A and inhibits its interaction with the cytokines IL-17A, IL-17F, IL-17C, IL-17A/F heterodimer and IL-25. Blocking IL-17RA and inhibiting the response induced by IL-17 cytokines and inhibiting the release of related pro-inflammatory factors.^[51]^ It was approved by the FDA in 2017, but in the process of using brodalumab, it was found that 6 patients in clinical trials committed suicide after receiving brodalumab treatment, and the FDA issued a safety warning on this, but there is still controversy about the correlation between inhibition of IL-17A and suicidal behavior.^[52]^

### 3.5. Netakimab

Netakimab (NTK) is a human monoclonal antibody targeting IL-17A. Eighty-nine patients with active AS were randomly assigned to receive subcutaneous NTK (40, 80, or 120 mg) or placebo for 16 weeks. The remission rates of ASAS20 were 72.73%, 81.82%, 90.91% and 42.86%, respectively. The study^[53]^ found that the fastest and safest effective dose was 120 mg, and the most common adverse reactions included lymphocytosis, neutropenia and asymptomatic bacteruria. In a double-blind, multicenter, randomized, placebo-controlled, phase 3 ASTERA study, 228 adult patients with AS received 120 mg subcutaneous injections of NTK or placebo at weeks 0, 1, and 2, and then every other week thereafter. At week 16, patients with CRP ≥ 5 mg/L had a higher risk of adverse events. Observed the AS patients with disease activity decline more apparent, CRP > 20 mg/L ASDAS - in patients with CRP and BASDAI improve the most obvious.^[54]^

## 4. The targeted IL-12/23 of the anti-rheumatism medicine

IL-23 is a heterodimeric cytokine, which is mainly produced by activated macrophages and dendritic cells. It plays an important role in inflammation, tumor immunity and autoimmune diseases. IL-23p19 and IL-12/IL-23p40 are 2 major subunits of IL-23. Both IL-23 and IL-12 can induce the differentiation of Th cells, and play an important role in the proliferation of Th17 cells and the maintenance of memory cells. It promotes Th17 cells to produce IL-17A, IL-17F, IL-22 and other cytokines, and promotes the expression of transcription factor STAT3, which is involved in the occurrence and development of a variety of diseases.^[55,56]^

IL-23/Th17 axis is an important inflammatory pathway in the pathogenesis of AS, and AS is closely related to IL-23 and IL-23R genes. The polymorphism of IL-23R gene is associated with AS, and the inflammation driven by IL-23 can lead to the formation of new bone in AS, which also reflects the possible involvement of IL-23 in the pathogenesis of AS.^[57]^ IL-23 is composed of p19 and p40 sub, p40 Shared with IL-12. Therefore, Ustekinumab (UST) that binds to the p40 subunit can inhibit both IL-12 and IL-23, and drugs that bind the p19 subunit, such AS Rizankizumab, Tildraki-zumab and Guselkumab, have recently been introduced into clinical studies for the treatment of AS.

### 4.1. Ustekinumab

UST is a fully human IgG1 monoclonal antibody targeting the p40 subunit of IL-12/23 interaction.^[58]^ In a 28-week prospective, open-label, proof-of-concept study, 20 patients with AS received 90 mg UST subcutaneously at baseline, week 4, and week 16. At week 24, 65% of patients achieved ASAS40 remission. 55% of the AS patients bath ankylosing spondylitis disease activity index (BASDAI) improve more than 50%.^[59]^ In addition, during the period of treatment, patients with MRI detected related inspection report the results of the parameters and active inflammation were improved significantly, and NSAIDs intake significantly reduced, clinical responses and the active inflammation of the MRI to reduce and reduce the related to the level of serum C-reactive protein UST safe and well tolerated.

### 4.2. Rizankizumab

IL-23 risankizumab targeted in p19 of humanized monoclonal antibody, in active for the treatment of patients with AS.^[60]^ In a phase 2 study,^[61]^ 159 patients with AS were randomized to different doses of risankizumab (18, 90, and 180 mg). At week 12, ASAS40 response rates were 25.5%, 20.5%, and 15.0% in the 18 mg, 90 mg, and 180 mg risankizumab groups, respectively, compared with 17.5% in the placebo group. risankizumab treatment did not meet the primary end point of the study, and compared with placebo, In patients with active AS there was no evidence shows that the improvement of the clinical significance. New evidence supports the efficacy and safety of TNFi and IL-17i in AS, while IL-23i has not shown a relevant effect, and observational studies are needed to further confirm its long-term safety.

## 5. Antirheumatic drugs targeting IL-1

**Figure 1.**
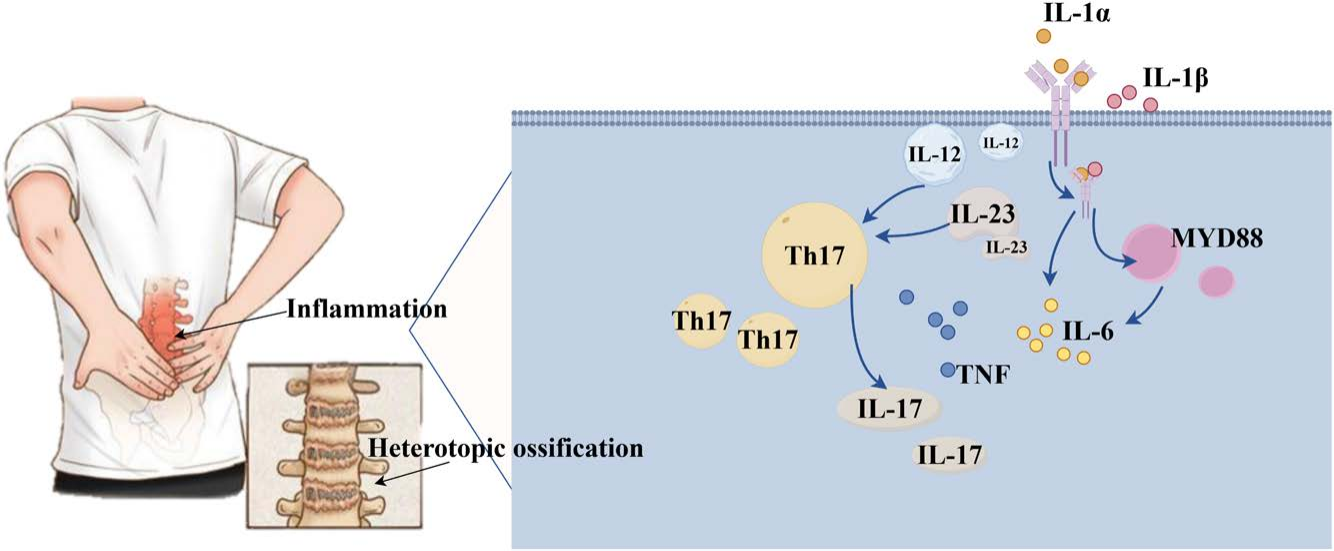
Chronic inflammation and heterotopic ossification are the 2 major characteristics of AS. Th17/IL-17 inflammatory axis plays an important role in the pathogenesis of AS. IL-17 can promote the release of IL-1, IL-6, IL-8, TNF and other inflammatory factors, IL-23 and IL-12 can induce the proliferation of Th17 cells. IL-1α or IL-1β combined with IL-1R recruited IL-1R3 to form a heterotrimer, which further formed MYD88 and promoted the expression of IL-6 and IFN. AS = ankylosing spondylitis, IFN = interferon, IL = interleukin, MYD88 = myeloid differentiation factor 88.

IL-1 is a inflammatory cytokines play an important role in, mainly for the IL-1 alpha and beta 2 IL-1. In healthy conditions, IL-1α can be expressed on the surface of the cell membrane, bind to the interleukin 1 receptor (IL-1R) of neighboring cells, or exist in the cytoplasm, and be released to play a role outside the cell when the cell is injured or necrotic. IL-1β is expressed in myeloid cells such as monocytes, macrophages and neutrophils in the form of inactive precursor. After caspase-1 cleavage and activation, it is secreted into the extracellular space in the active form to combine with IL-1R to play a role.^[62]^ After combining with IL-1R, IL-1α or IL-1β recruited IL-1R3 to form a heterotrimer, which further recruited myeloid differentiation factor 88 (MYD88) and activated a variety of transcription factors such as NF-κB and mitogen-activated protein kinase through phosphorylation cascade.^[63]^ Activation of these transcription factors promotes interleukin 6 (IL-6), Interferon (IFN) and other cytokines, expression of endothelial cell adhesion factors, vasodilation, and proliferation and differentiation of helper T and B lymphocytes^[64]^ (Fig. 1).

Anakinra is an adequate non-glycosylated IL-1R antagonist that blocks the binding of IL-1α and IL-1β to the receptor and was approved by the FDA in 2001.^[65]^ In a trial evaluating the safety and efficacy of anakinra in the treatment of AS,^[66]^ 20 patients with AS refractory to nonsteroidal anti-inflammatory drugs received subcutaneous injection of 100 mg anakinra daily for 24 weeks. After 24 weeks, BASDAI scores were assessed and ASAS was assessed with ankylosing spondylitis. Studies have found that anakinra can only improve the spinal symptoms of a small proportion of AS patients, and injection site reactions are the most common adverse reactions. In another group that had the element of the open experiment, 9 active AS to significant improvement in patients with inflammatory reaction, inflammation of the spine on MRI attachment points have been obviously improved.^[67]^

## 6. Antirheumatic drugs targeting IL-6

IL-6 is involved in both pro-inflammatory and anti-inflammatory pathways, which is rapidly produced during infection and tissue injury and stimulates the inflammatory response through the acute phase. Although the expression of IL-6 is strictly controlled by transcriptional and posttranscriptional mechanisms, persistent dysregulation of IL-6 can still lead to the occurrence of chronic inflammation and the formation of a variety of autoimmune system diseases.^[68]^ Tocilizumab (TXZ) AS the first synthetic receptors on IL-6 monoclonal antibody, is currently the only clinically used AS a treatment of IL-6 antibody.^[69]^ A multicenter, randomized controlled trial in patients with active AS showed no significant change in BASDAI in the TXZ group compared with the placebo group.^[70]^ After 6 months of TXZ treatment, the CRP and BASDAI scores of 4 AS patients with peripheral synovitis were significantly lower than those before treatment.

Koumakis^[71]^ found that compared with the spinal MRI of AS patients before and after treatment, the inflammatory signal of the spine of AS patients was significantly relieved after TXZ treatment. TXZ not only reduced the CRP level and clinical symptom score, but also delayed the imaging progression of AS patients. The efficacy of TXZ in the treatment of AS is still controversial, so a large number of clinical studies are needed to verify its safety and efficacy. In the BUILDER-1 and BUILDER-2 studies to evaluate the short-term efficacy and safety of Tocilizumab, 102 patients with AS were included, and C-reactive protein levels decreased during the process, suggesting that IL-6 receptor was sufficiently blocked, and hypersensitivity and hypersensitivity reactions should be vigilant during the use of TCZ.^[70]^

## 7. Antirheumatic drugs targeting leukocyte differentiation antigens

Rituximab (RTX) is a monoclonal antibody directed against the B cell surface molecule CD20.^[72]^ Study found that RTX in TNF alpha treatment failure after the AS patients showed good treatment effect, and can be AS a response to TNFi lose RTX patients or entity potential target therapy for patients with malignant tumor.^[73]^ In the 1-year follow-up, 45% of AS patients initially treated with RTX showed a good clinical response at the end of the first year. The potential role of RTX in the treatment of AS is still unclear, and larger controlled studies are needed to evaluate the role of RTX in the treatment of active AS patients in the future.^[74]^

## 8. Conclusions and prospects

AS a representative of targeted therapy, biological agents have shown outstanding advantages in the treatment of AS. This article through to the targeted TNF alpha, targeted IL/17, targeted IL-12/23, targeted, targeted IL-6 and IL-1 targeting leukocyte differentiation antigen anti-rheumatism medicine on the clinical trials of summary, clear of advantages and disadvantages of different types of drugs in the treatment process, help us to determine the optimal dosage regimen in the future (Table 2). At present, the axSpA treatment guidelines published by APLAR in 2018 recommend TNFi AS the initial treatment drug for AS patients with biological agents. For adult patients with AS who still have disease activity after at least 12 weeks of adequate TNFi treatment, another TNFi or Secukinumab treatment is conditionally recommended.^[75]^ Subsequent guidelines^[76]^ state that TNFi, Secukinumab, or IXE are strongly recommended for patients with active AS after NSAIDs treatment, and that the level of evidence recommending TNFi over Secukinumab or IXE is very low.

**Table 2:** Summary of clinical trials related to ankylosing spondylitis.

Type	Drugs	Clinical outcomes
Antirheumatic drugs targeting TNF-α	Etanercept	Four mo after treatment, Etanercept group, 80% of patients have a response to treatment and placebo groups only 30% of the patients have the response to treatment (*P* = .004).^[12]^ Compared with the control group, the proportion of patients with reduced sacroiliac joint erosion and increased backfilling under MRI in the Etanercept group was significantly higher. ASDAS continued after the use of Etanercept was 32.7% (34/104) of patients in remission and didn’t get ASDAS compared to continue to ease the patients (5/104), erosion decrease and the proportion of backfilling increase significantly higher (*P* < .001).^[13]^
	Infliximab (IFX)	After 24 wk IFX treatment, IFX group has 61.2% of the AS patients achieve remission, compared to 19.2% of placebo patients (*P* < .001).^[17]^
	Adalimumab (ADA)	At week 12, 58.2% of AS patients treated with ADA40 mg subcutaneously every other week achieved clinical remission, compared with 20.6% of those treated with placebo (*P* < .001).^[23]^
	Golimumab (GDM)	Compared with the placebo group, the remission rate of ASAS20 in the GDM group was 71.1%, which was significantly higher than the clinical remission rate of 40.0% in the placebo group (*P* < .0001). The remission rate of ASAS40 in the GDM group was 56.7%, which was significantly higher than the 23.0% in the placebo group (*P* < .0001).^[32]^
	Certolizumab pegol (CZP)	CZP could rapidly reduce the symptoms and signs of AS. The ASAS20 remission rate was 57.7% in CZP200 mg every 2 wk group, 63.6% in CZP400 mg every 4 wk group, and 38.3% in placebo group (*P* < .001).^[36]^
Antirheumatic drugs targeting IL-17	Secukinumab	Compared with placebo, the improvement in BASDAI was statistically significant in group I and group II (−2.3 vs −0.6; *P* < .0001).^[43]^
	Ixekizumab (IXE)	At week 16, 18% of placebo patients and 52% of IXEQ2 patients achieved ASAS40 (*P* < .0001).^[46]^
	Bimekizumab	Every 4 wk to patients with medicine Bimekizumab160 mg AS ASAS40 was achieved at week 16 patients (47.7%), only 21.4% of the placebo group of the AS patients achieve ASAS40 (*P* < .001).^[49]^
	Brodalumab	FDA safety warning after 6 patients in clinical trials committed suicide after receiving Brodalumab.^[52]^
	Netakimab (NTK)	The study found that the effect to the fastest and most secure effective dose of 120 mg, the most common adverse reactions include disease of grow in quantity of lymphocytes, neutropenia and asymptomatic bacteriuria.^[53]^
Antirheumatic drugs targeting IL-12/23	Ustekinumab (UST)	20 patients with AS received 90 mg UST subcutaneously at baseline, week 4, and week 16. At week 24, 65% of patients achieved ASAS40 remission, and 55% of patients with AS had more than 50% improvement in BASDAI.^[59]^
	Risankizumab	At week 12, the ASAS40 response rates were 25.5%, 20.5%, and 15.0% in the 18 mg, 90 mg, and 180 mg risankizumab groups, respectively, compared with 17.5% in the placebo group. risankizumab treatment did not meet the primary end point of the study, and compared with placebo, no evidence of clinically meaningful improvement has been shown in patients with active AS.^[61]^
Antirheumatic drugs targeting IL-1	Anakinra	After 24 wk of subcutaneous injection of anakinra 100 mg daily, BASDAI scores of patients were evaluated, and ASAS was evaluated in ankylosing spondylitis. The study found that anakinra improved spinal symptoms in only a small proportion of patients with AS, and injection site reactions were the most common adverse reactions.^[66]^
Antirheumatic drugs targeting IL-6	Tocilizumab (TXZ)	TXZ AS after treatment in patients with spinal signal obviously alleviate inflammation, TXZ lower CRP levels and clinical symptom scores, not only can delay the AS radiographic progression in patients with.^[71]^
Antirheumatic drugs targeting leukocyte differentiation antigens	Rituximab (RTX)	Found in the period of 1-yr of follow-up, in patients with RTX treated AS initially, 45% of the patients showed good clinical response at the end of the first, but the underlying mechanism is unclear.^[74]^

**Figure 2.**
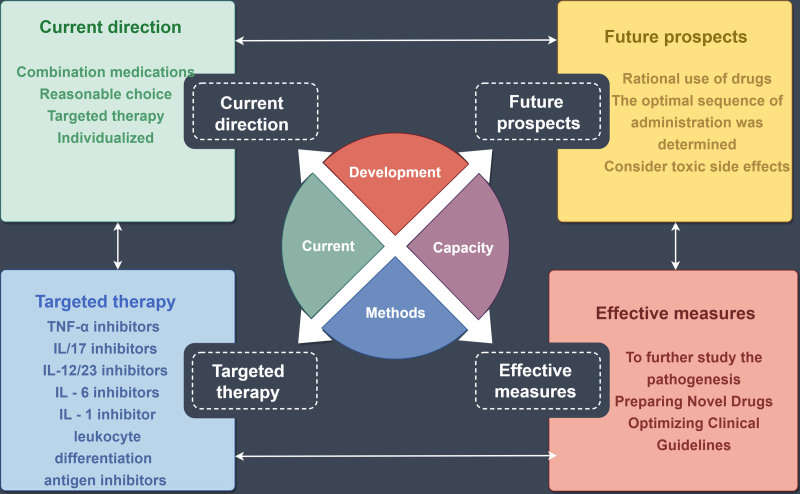
The targeted therapy of AS is still mainly targeting TNF-α, IL-17, IL-12/23, IL-1, IL-6 and leukocyte differentiation antigen drugs, but considering the adverse drug reactions and other issues, we should rationally use targeted drugs to develop a safer and more effective drug administration regimen. AS = ankylosing spondylitis, IL = interleukin, TNF-α = tumor necrosis factor-α.

The efficacy and toxic side effects of biological agents coexist, and the adverse reactions and side effects accompanying the use of biological agents are also problems that we need to consider, such as infection, allergy, adverse reactions at the injection site, increasing the risk of cancer and inducing tuberculosis. AS treatment choice despite the varied, but there are still a response to the current drugs in patients with such problems AS inadequate or intolerance, this needs us to consider early treatment at the same time, the reasonable choice of the AS targeted drugs. In the selection of drugs, the best order of administration, the switch between drugs and possible combination therapy can be further studied. New drug combination methods still need to be supported by a large number of clinical research data. It is believed that in the future, more large-scale and multicenter clinical studies will provide reasonable data for the clinical use of AS. In the future, the continuous development of molecular biology, the in-depth research on the pathogenesis of AS, the emergence of new drugs, and the further optimization of clinical diagnosis and treatment guidelines will provide more options for the treatment of AS. We believe that AS patients will be treated more systematically and safely in the future (Fig. 2).

## Author contributions

**Conceptualization:** Jiapeng Wang, Wang Lou, Yingnan Li.

**Data curation:** Jiapeng Wang, Wang Lou, Yingnan Li, Yang Jiang, Xue Jiang.

**Formal analysis:** Lin Yang.

**Investigation:** Xue Jiang, Lin Yang.

**Validation:** Yang Jiang.

**Writing – original draft:** Jiapeng Wang.

**Writing – review & editing:** Jiapeng Wang.
